# Serum cytokine profiling reveals different immune response patterns during general and severe *Mycoplasma pneumoniae* pneumonia

**DOI:** 10.3389/fimmu.2022.1088725

**Published:** 2022-12-22

**Authors:** Zhikun Zhang, Haiwei Dou, Peng Tu, Dawei Shi, Ran Wei, Ruijie Wan, Chunmei Jia, Lihua Ning, Dongmei Wang, Jing Li, Yan Dong, Deli Xin, Baoping Xu

**Affiliations:** ^1^ Beijing Tropical Medicine Research Institute, Beijing Friendship Hospital, Capital Medical University, Beijing, China; ^2^ Department of Pediatric, Baotou Fourth Hospital, Baotou, Inner Mongolia, China; ^3^ Department of Pediatric, Beijing Chang Ping District Hospital of Traditional Chinese Medicine and Western Medicine, Beijing, China; ^4^ Department of Respiratory, Beijing Children’s Hospital, Capital Medical University, Beijing, China

**Keywords:** *Mycoplasma pneumoniae*, cytokines, immune status, immune response, severe MPP, general MPP

## Abstract

*Mycoplasma pneumoniae* (MP) is an important human pathogen that mainly affects children causing general and severe *Mycoplasma pneumoniae* pneumonia (G/SMPP). In the present study, a comprehensive immune response data (33 cytokines) was obtained in school-age children (3–9 years old) during MPP, aiming to analyze the immune response patterns during MPP. At acute phase, changes of cytokines were both detected in GMPP (24/33) and SMPP (23/33) groups compared to the healthy group (p < 0.05), with 20 identical cytokines. Between MPP groups, the levels of 13 cytokines (IL-2, IL-10, IL-11, IL-12, IL-20, IL-28A, IL-32, IL-35, IFN-α2, IFN-γ, IFN-β, BAFF, and TSLP) were higher and three cytokines (LIGHT, OPN and CHI3L1) were lower in the SMPP group than in the GMPP group (p < 0.05). Function analysis reveals that macrophage function (sCD163, CHI3L1) are not activated in both MPP groups; difference in regulatory patterns of T cells (IL26, IL27, OPN, LIGHT) and defective activation of B cells (BAFF) were detected in the SMPP group compared to the GMPP group. Besides, the level of osteocalcin; sIL-6Rβ and MMP-2 are both decreased in MPP groups at acute and convalescent phases compared to the healthy group, among which the levels of sIL-6Rβ and MMP-2 showed negative correlations (p < 0.1) to the application of bronchial lavage in SMPP group, indicating their roles in the development of MPP. At the convalescent phase, more cytokines recovered in GMPP (18) than SMPP (11), revealing better controlled immune response during GMPP. These results reveal different immune response patterns during GMPP and SMPP. In addition, the differentiated cytokines may serve as potential indicators of SMPP; early intervention on immune response regulations may be helpful in reducing the severity of SMPP.

## 1 Introduction

*Mycoplasma pneumoniae* (MP), the smallest prokaryotic pathogenic microorganism, is an important pathogen of community-acquired pneumonia (CAP) (1). *Mycoplasma pneumoniae* pneumonia (MPP) processes a higher incidence rate in school-age children than in adults, which can be classified into general MPP (GMPP) and severe MPP (SMPP) according to the severity of the disease (2). The age distribution of MPP indicates a potential defective immune response to MP infection in school-age children (3). However, the pathogenesis of MPP in children is not fully understood.

The pathogenesis of MP mainly includes direct damages and immune damages, which are highly correlated (4, 5). MP can adhere to the host cell membrane *via* the apical structure. This process is helpful in nutrient acquisition and avoiding respiratory clearance. MP lipoproteins can be recognized by toll-like receptor (TLR) heterodimers (TLR2/6 and TLR2/1) (6), resulting in up-regulation of pro-inflammatory cytokines such as TNF-α, IL-1β, IL-4, IL-6, and IL-8 (7, 8). The induction of pro-inflammatory cytokines can stimulate a distinctive innate immune response in the lung (9). In the bronchoalveolar lavage fluid (BALF) of MPP, the levels of IL-1β, IL-2, IL-4, IL-6, IL-8, and IL-10 were shown to be up-regulated (10, 11). Cytokines changes in serum were also detected, including IL-1β, IL-2, IL-5, IL-6, IL-12, IL-18, IFN-γ, and TNF-α (12). Several cytokines were reported to correlate with the severity of MPP (12), but in most studies only few cytokines were examined, which makes it difficult to analyze the immune response patterns of the host.

A common feature of MPP is the infiltration of inflammatory cells into the lungs (13). In BALF, the proportion of neutrophils was higher in SMPP than in GMPP (14), which may relate to a higher amount of MP, since the level of MP correlates with the level of IL-8 in lung (15). In addition, cytokine analysis of BALF also revealed a differential regulation of T cell subsets during MPP. A higher ratio of Th2/Th1 (IL-4/IFN-γ) cells in SMPP and a higher proportion of Th17 (IL-17) cells in refractory MPP were detected than in GMPP (16, 17). On the other hand, although MP was reported as a B cell activator, variable response of antibodies has been reported in clinical cases (18), just as serological tests (especially IgA and IgG) gain lower diagnostic values than the real-time PCR method at acute phase of MPP (19). Meanwhile, macrophages can be activated during MP infection by MyD88 pathway and play important roles in the clearance MP in a mouse model (20). However, the ratio of macrophages in the lung decreased with the severity of MPP (21), suggesting that the activation of macrophage is hampered during MP infection, which may be associated with restricted migration or altered gene expression of macrophages during MPP (22, 23). The activation modes of the B cell and macrophage may relate to the cellular structure and infection mode of MP. Their relationship to the development of MPP needs to be clarified.

In recent years, the isolation rate of macrolide-resistant (MR) strains of MP has gradually increased, which increases the difficulty in treatment of SMPP (24). The limitation of antibiotics emphasizes the importance of analyzing the immune response patterns during MPP (25). In this study, cytokine analysis reveals different immune responses of T-cells, B-cells, and macrophage during GMPP and SMPP, which are helpful in understanding the pathogenesis of MPP.

## 2 Materials and methods

### 2.1 Patients and controls

Patients with SMPP or GMPP were recruited from Beijing Children’s Hospital and Baotou fourth hospital. Diagnosis of MPP was conducted following the Chinese Medical Association guidelines for management of CAP in children (26): (1) fever (>37.3°C) or acute respiratory symptoms or both; (2) decreased breath sounds, dry wet rales; (3) chest radiograph showed at least one of the following: spotted or patchy immersion; interstitial changes; lobar parenchymal infiltration shadow; enlargement of hilar lymph nodes; (4) positive PCR results or ≥4-fold seroconversion of MP antibody titer. SMPP was defined as MPP with one of the following: (1) poor general condition; (2) increased breathing rate; (3) cyanosis and dyspnea; (4) multi-lobed or ≥2/3 infiltration of the lung; (5) transcutaneous oxygen saturation ≤92% in room air; (6) extrapulmonary complications. Samples of healthy group were enrolled from the ones underwent a health checkup in Beijing Changping Hospital of integrated traditional Chinese and Western Medicine, with the standard as: (1) no symptoms of respiratory tract infections within 1 month; (2) not being diagnosed as MP infections within 6 months; (3) diagnosed as health in the checkup. All the participants should follow the standards: (1) no dysplasia and malnutrition; (2) no diseases relating to immune system, cardiovascular, liver, kidney and hematopoietic system; (3) signed informed consent.

### 2.2 Sample collection

The acute phase samples were collected within 48h post being diagnosed as GMPP or SMPP. All the acute phase samples were collected before treatment of glucocorticoid and azithromycin. The convalescent phase samples were collected within 72h when no fever or improvement of respiratory symptoms. All the samples were placed at room temperature for about 2h, serum were isolated by centrifugation, transferred to a new tube and stored at -80°C.

### 2.3 Molecular detection of MP

Throat swabs were collected from the patients and soaked in PBS or SP4 medium. DNAs of the samples were isolated using the DNA Extraction Kit (CWbio, China). Primers of 23SrRNA (F:5-GACACCCGTTAGGCGCAA-3, R:5-CTGGATAACAGTTACCAATTAGAACAGC-3) were applied in qPCR to detect the existence of MP (27). A PCR program was conducted on the 7500fast real-time PCR system (ABI, USA) using the UltraSYBR Mixture (CWbio, China). The PCR products were sequenced (Sangon, China) and analyzed to the sequence of 23SrRNA in standard strain (M129) to detect macrolide-resistant related mutations.

### 2.4 Detection of cytokines

All serum samples were applied to the cytokine detection kit (Bio-Plex Pro™ Human Inflammation Panel 1, 37-Plex, Bio-Rad) for the levels of cytokines. The experiment and adjustment of instrument were conducted following manufacturer instructions (28); the results were obtained by Milliplex Analyzer (Luminex 200) (**Table S1**). Both the samples, quality controls and standards, were detected in two duplicates. The levels of cytokines were calculated to corresponding standard curves. The data 50% above maximum concentration of standard samples was not applied for statistical analysis.

### 2.5 Statistical analysis

All analyses were performed using SPSS Statistics (version 20; IBM Corporation, Armonk, NY, USA) for Windows. One-way analysis of variance was conducted and expressed as mean ± standard deviation. Normally distributed variables were applied in Dunnett’s *t*-test for comparison between groups; abnormally distributed variables were compared with Mann–Whitney test, and a probability (p) value of <0.05 was considered statistically significant. Pearson correlation analyses between bronchial lavage and cytokines were determined by bivariate correlation analysis; a probability (p) value of <0.1 was considered statistically significant.

## 3 Results

### 3.1 General information

A total of 36 participants were involved in this study, including 12 healthy children, 11 children with GMPP, and 13 children with SMPP; the information of participants is listed in **Table 1**. No significant differences in age and gender distribution between groups were obtained. A longer duration of fever (p < 0.05) and higher rate of bronchial lavage were observed in the SMPP group. Sequence analysis reveals similar macrolide resistance (MR) ratios of 91% (10/11) in the GMPP group and 92.3% (12/13) in SMPP group. Routine blood test showed higher levels of white blood cell count, platelet count, and lymphocyte ratio in the GMPP group (**Table 1**), while SMPP group has a higher level of CRP and neutrophil ratio in the SMPP group, but these difference were not statistically different (p > 0.05) between MPP groups (**Table 1**).

**Table 1:** General information of the participants: Statistical differences between GMPP and SMPP groups were marked with * on item names.

Groups	Control	GMPP	SMPP
Age (mean ± STD)	6.33 ± 1.75	6.48 ± 1.69	7.12 ± 1.79
Gender (male/female)	8/4	7/4	8/5
MR rate	–	91%	92.3%
Bronchial lavage (yes/no)	–	0/11	7/6
*Duration of fever (day)	–	4.88 ± 0.92	6.08 ± 1.19
Leukocyte count (×10^9^ cells/L)	–	7.04 ± 2.22	7.34 ± 2.84
Platelet count (×10^9^ cells/L)	–	237.82 ± 56.2	333.08 ± 157.98
Neutrophil count (%)	–	57.43 ± 10.93	66.92 ± 13.05
Lymphocyte count (%)	–	30.07 ± 10.89	23.12 ± 12.09
CRP (mg/L)	–	12.44 ± 11.18	19.44 ± 14.37

### 3.2 Cytokine profiling analysis of GMPP and SMPP

The levels of 33 cytokines were successfully obtained; four cytokines (IL-12 (p70), IL-19, IL-22 and IL-34) with detection rates less than 50% in all groups and phases were not used for statistical analysis (**Table S1**). To give a general view of the immune response patterns during MPP, cytokines that changed significantly (p < 0.05) between groups and phases were analyzed.

#### 3.2.1 Cytokine profiling at acute phase

**Figure 1:**
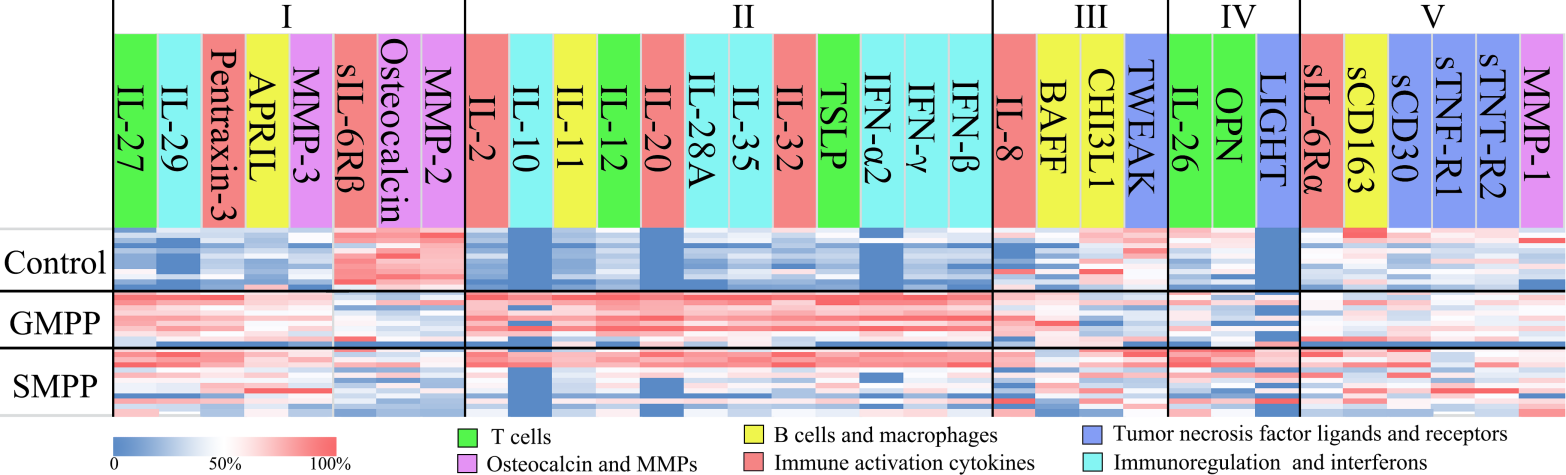
Cytokine profiles in the GMPP, SMPP, and Control groups. The cytokines were clustered by statistical analysis between groups and colored by their main deduced functions. Clusters I and II represent the cytokines that significant regulated in both MPP groups compared to control group, the cytokines were divided into the ones with (II) or with no (I) significant differences (p < 0.05) between MPP groups; Cluster III represent the cytokines that significant regulated in GMPP but not SMPP group compared to control group; Cluster IV represent the cytokines that significant regulated in SMPP but not GMPP group compared to control group; Cluster V represent the cytokines that did not show significant differences between groups (p < 0.05).

Compared to the healthy group, a total of 24 cytokines were changed in the GMPP group; 23 cytokines were changed in SMPP group significantly (p < 0.05) at acute phase, 20 of which are identical between MPP groups including: IL-2, IL-10, IL-11, IL-12, IL-20, IL-27, IL-28A, IL-29, IL-32, IL-35, TSLP, IFN-α2, IFN-γ, IFN-β, APRIL MMP-3, Pentraxin-3, Osteocalcin↓, MMP-2↓, and sIL-6Rβ↓. Four cytokines (IL-8, BAFF, TWEAK↓, CHI3L1↓) were only altered in GMPP group, and three cytokines (IL-26, OPN, LIGHT) were only altered in SMPP group (**Figure 1**; **Table S1**). The massive identical cytokines suggest both extensive immune responses during GMPP and SMPP.

**Figure 2:**
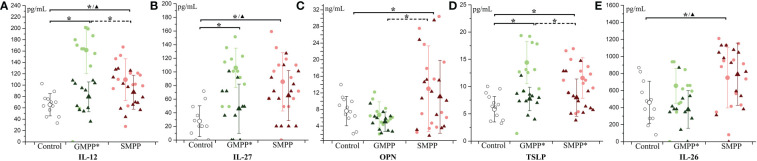
The level of T cell-related cytokines during MPP: **(A)** IL-12; **(B)** IL-27; **(C)** OPN; **(D)** TSLP; **(E)** IL-26. The healthy group (Control) is indicated with hollow circle; MPP groups are indicated with light circle (acute phase) and dark triangle (convalescent phase) of different colors (green: GMPP; red: SMPP). Mean values and standard deviations (STD) were represented with larger symbols. Significant differences (p < 0.05) were indicated with solid line (to the control group) or dotted line (between MPP groups) marked with * represents the acute phase, ^▲^ represents the convalescent phase. Significant differences (p < 0.05) between acute phase and convalescent phase were marked with * on group names.

**Figure 3:**
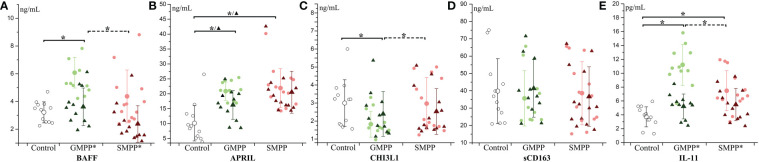
The level of B cell and macrophage related cytokines: **(A)** BAFF; **(B)** APRIL; **(C)** CHI3L1; **(D)** sCD163; **(E)** IL-11. Indications of groups and symbols are the same as described in **Figure 2**.

**Figure 4:**
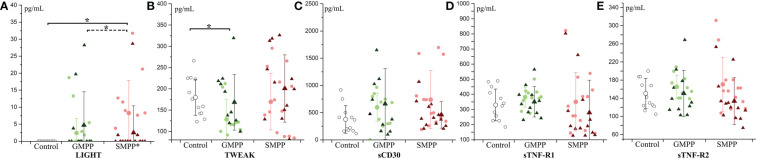
The level of tumor necrosis factor ligands and receptors: **(A)** LIGHT; **(B)** TWEAK; **(C)** sCD30; **(D)** sTNF-R1; **(E)** sTNF-R2. Indications of groups and symbols are the same as described in **Figure 2**.

**Figure 5:**
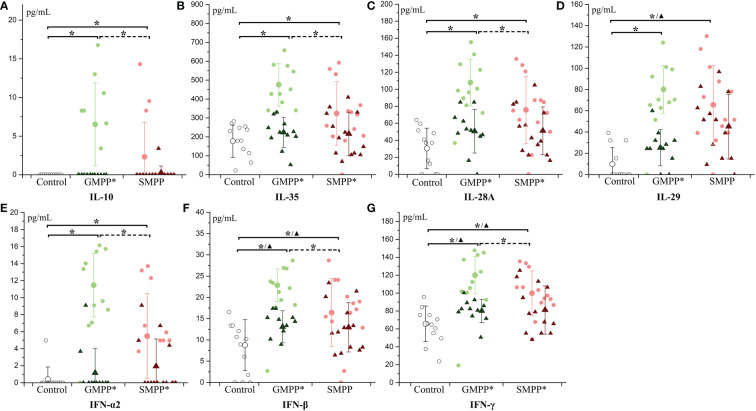
The level of immunoregulation cytokines and interferon: **(A)** IL-10; **(B)** IL-35; **(C)** IL-28A; **(D)** IL-29; **(E)** IFN-α2; **(F)** IFN-β; **(G)** IFN-γ. Indications of groups and symbols are the same as described in **Figure 2**.

**Figure 6:**
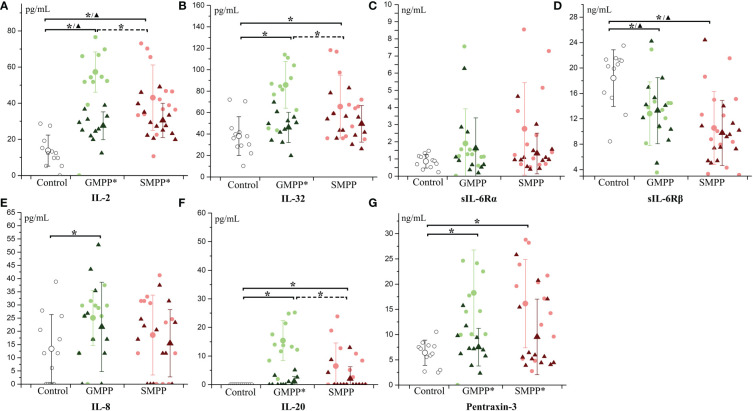
The level of multifunctional cytokines: **(A)** IL-2; **(B)** IL-32; **(C)** sIL-6Rα; **(D)** sIL-6Rβ; **(E)** IL-8; **(F)** IL-20; **(G)** Pentraxin-3. Indications of groups and symbols are the same as described in **Figure 2**.

**Figure 7:**
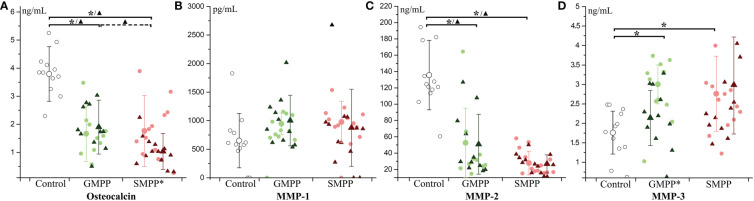
The level of Osteocalcin and matrix metalloproteinases (MMPs): **(A)** Osteocalcin; **(B)** MMP-1; **(C)** MMP-2; **(D)** MMP-3. Indications of groups and symbols are the same as described in **Figure 2**.

At acute phase, 16 cytokines were different between GMPP and SMPP groups (p < 0.05), 13 (IL-2, IL-10, IL-11, IL-12, IL-20, IL-28A, IL-32, IL-35, TSLP, BAFF, IFN-α2, IFN-β, and IFN-γ) of which were lower, and three (LIGHT, OPN, and CHI3L1) of which were higher in the SMPP group. These suggest differences in immune responses between GMPP and SMPP at acute phase (**Figures 1**–**7**; **Table S1**). Function analysis of these cytokines reveals different immune response patterns during GMPP and SMPP at acute phase: i) Both cellular and humoral immune responses are activated during GMPP, except for the decrease in macrophage function (CHI3L1) and apoptosis (TWEAK). ii). The immune response, immunoregulation cytokines, and interferon (IFN) are elevated in the SMPP but lower than in the GMPP group. iii) Difference in regulation of T cell (OPN, IL-26), increased apoptosis (LIGHT), and defective activation of B cell (BAFF) were detected in SMPP (**Figure 1**).

#### 3.2.2 Cytokine profiling at convalescent phase

In the GMPP group 18 cytokines (IL-2, IL-10, IL-11, IL-12, IL-20, IL-26, IL-27, IL-28A, IL-29, IL-32, IL-35, Pentraxin-3, TSLP, IFN-α2, IFN-γ, IFN-β, BAFF, and MMP-3) were decreased at the convalescent phase compared to the acute phase, and all these cytokines were elevated at the acute phase (p < 0.05). Seven cytokines (IL-2, APRIL, IFN-γ, IFN-β, sIL-6Rβ↓, MMP-2↓, and Osteocalcin↓) remained showing difference in the healthy group (p < 0.05) (**Figures 2**–**7**; **Table S1**). In the SMPP group, 11 cytokines (IL-2, IL-11, IL-35, IL-28A, TSLP, IFN-α2, IFN-γ, LIGHT, BAFF, Osteocalcin, Pentraxin-3) were decreased at the convalescent phase compared to acute phase (p < 0.05), with BAFF unchanged and Osteocalcin decreased at the acute phase. Eleven cytokines remained showing difference in the healthy group (p < 0.05) (**Figures 2**–**7**; **Table S1**), containing the seven differentiated cytokines in the GMPP group at convalescent phase and four extra cytokines (IL-12, IL-26, IL-27, IL-29). Only osteocalcin was significantly different between the GMPP and SMPP groups at convalescent phase. Among the seven identical cytokines between the GMPP and SMPP groups at convalescent phase, low levels of sIL-6Rβ, MMP-2, and osteocalcin were significantly decreased at the acute and convalescent phases in both MPP groups, and the levels of osteocalcin and sIL-6Rβ were further reduced at the convalescent phase in the SMPP group, suggesting these cytokines are important features of MPP and may relate to the severity of MPP.

### 3.3 Immune response analysis during GMPP and SMPP

Further, the cytokines were clustered into six groups with their main deduced functions. The levels of the same cytokine in all groups and phases were displayed in the same figure to exhibit the difference in cytokine regulations between GMPP and SMPP.

#### 3.3.1 T cell related cytokines

IL-12 and IL-27, which related to the differentiation of Th1 cells, increased at the acute phase (p < 0.05) in the GMPP and SMPP groups, and IL-12 in the GMPP group was higher than that in the SMPP group (p < 0.05). At the convalescent phase, the levels of IL-12 and IL-27 in the GMPP group reduced (compared with acute phase, p < 0.05) to the similar level of the healthy group; the levels of IL-12 and IL-27 in the SMPP group were still higher than in the healthy group (p < 0.05) (**Figures 2A, B**). OPN, involved in the upregulation of IL-12, was significantly higher in the SMPP group than in the GMPP and healthy groups at the acute phase (**Figure 2C**). TSLP, relating to the differentiation of Th2 cells, increased in the GMPP and SMPP groups at the acute phase (p < 0.05), and it was higher in the GMPP group than in the SMPP group (p < 0.05). TSLP significantly decreased (compared with the acute phase, p < 0.05) in the GMPP and SMPP groups to the similar level of the healthy group at the convalescent phase (**Figure 2D**). IL-26, relating to the differentiation of Th17 cells, was significantly higher in the SMPP group than in the healthy group at the acute and convalescent phases (p < 0.05) (**Figure 2E**). These suggest that T cell subsets are differently regulated during GMPP and SMPP.

#### 3.3.2 B cell and macrophage-related cytokines

The B cell activator BAFF was only significantly elevated (p < 0.05) in the GMPP group compared to the healthy group at the acute phase. BAFF decreased in the GMPP and SMPP group at the convalescent phase compared to the acute phase (**Figure 3A**) (p < 0.05). APRIL relating to the development of the B cell was significantly higher in the GMPP and SMPP groups than in the healthy group at both acute and convalescent phases (**Figure 3B**) (p < 0.05). CHI3L1 relating to macrophage function was lower in the acute phase in the GMPP group than in the control and SMPP groups (**Figure 3C**) (p < 0.05). No significant differences were detected between groups in sCD163, which relates to the activation of the macrophage (**Figure 3D**). IL-11, which promotes the proliferation of both B cells and macrophages, increased in both MPP groups at the acute phase and was higher in the GMPP group than in the SMPP group (p < 0.05). IL-11 decreased (to the similar levels of healthy group) in both MPP groups at the convalescent phase (**Figure 3E**) (p < 0.05). These suggest potential defect in the activation of macrophage functions and B cells at the acute phase of GMPP and SMPP.

#### 3.3.3 Tumor necrosis factor ligands and receptors

LIGHT, a ligand for TNFRSF14, was significantly increased in the SMPP group at the acute phase, and decreased (compared with the acute phase, p < 0.05) to the similar level of the healthy group at the convalescent phase (**Figure 4A**). TWEAK, a ligand for the FN14/TWEAKR receptor, was significantly decreased in the GMPP group at the acute phase to the healthy group (p < 0.05), and a higher level of TWEAK was detected in the SMPP group than in the GMPP group at the acute phase (p = 0.085) (**Figure 4B**). The other three tumor necrosis factor receptor-related cytokines (sTNF-R2, sTNF-R1 and sCD30) were not significantly changed in both MPP groups (**Figures 4C–E**).

#### 3.3.4 Immunoregulation cytokines and interferons

IL-10 and IL-35, relating to immunoregulation, both increased at the acute phase in MPP groups (p < 0.05), and they were higher in the GMPP group than in the SMPP group (p < 0.05). At the convalescent phase, IL-10 decreased in the GMPP group; IL-35 decreased in both MPP groups (p < 0.05). No significant differences of IL-10 and IL-35 between groups were detected at the convalescent phase (**Figures 5A, B**). All five interferons increased at the acute phase in both MPP groups (p < 0.05), and IL-28A (IFNλ2), IFN-α2, IFN-β, and IFN-γ were higher in the GMPP group than in the SMPP group (p < 0.05). All five interferons decreased (compared with acute phase) in the GMPP group at the convalescent phase (p < 0.05), IFN-β and IFN-γ were still higher in GMPP groups than in the healthy group at the convalescent phase (p < 0.05); IFN-α2, IFN-γ, and IL-28A (IFNλ2) decreased in the SMPP group (compared with acute phase) at the convalescent phase; IL-29 (IFNλ1), IFN-β, and IFN-γ were still higher in the SMPP group than in the healthy group at the convalescent phase (p < 0.05) (**Figures 5C–G**). These indicate that immunoregulation cytokines and interferons are both activated during MPPs, and they are higher in GMPP than SMPP at the acute phase.

#### 3.3.5 Other interleukin and related cytokines

IL-2 and IL-32, relating to the activation of a variety of immune cells, increased at the acute phase and were higher in the GMPP group than in the SMPP group. At the convalescent phase (p < 0.05), IL-2 decreased in both the GMPP and SMPP groups (compared with acute phase, p < 0.05); IL-2 was still higher than in the healthy group (**Figure 6A**). At the convalescent phase, IL-32 decreased (compared with the acute phase, p < 0.05) in the GMPP group; no significant difference was detected between groups (**Figure 6B**). sIL-6Rα, a part of the receptor complex of IL-6, was not significantly regulated in both groups (**Figure 6C**). sIL-6Rβ, a part of the receptor complex of IL-6 and several other cytokines, decreased in both the acute and convalescent phases in the GMPP and SMPP groups (p < 0.05), and sIL-6Rβ was higher in the GMPP group than in the SMPP group at the convalescent phase (p = 0.056) (**Figure 6D**). IL-8, relating to neutrophil function, increased in the GMPP group compared to the healthy group at the acute phase (p < 0.05) (**Figure 6E**). IL-20, relating to skin inflammation, increased in the GMPP and SMPP groups at the acute phase, and it was higher in the GMPP group than in the SMPP group; At the convalescent phase, IL-20 in both MPP groups reduced to the similar level of the healthy group, and this reduction was significant in the GMPP group (p < 0.05) (**Figure 6F**). Pentraxin-3, involved in regulating inflammation and complement activation, increased in both the GMPP and SMPP groups at the acute phase, and decreased (compared with acute phase, p < 0.05) in both MPP groups to a similar level of the healthy group at the convalescent phase (**Figure 6G**). These suggest stronger immune response in GMPP than in SMPP at the acute phase; the function of sIL-6Rβ is impaired during MPP.

#### 3.3.6 Osteocalcin and matrix metalloproteinases

Osteocalcin, relating to the activity of osteoblasts, decreased at the acute and convalescent phases in both MPP groups (p < 0.05), and it was lower in the SMPP group than in the GMPP group at the convalescent phase (p < 0.05) (**Figure 7A**). The MPPs are both involved in the breakdown of extracellular matrix in normal physiological processes. The level of MMP-1 was not significantly influenced in both MPP groups (**Figure 7B**). MMP-2 decreased at the acute and convalescent phases in both MPP groups (**Figure 7C**) (p < 0.05). MMP-3 increased in both MPP groups at the acute phase (**Figure 7D**) (p < 0.05), and decreased (compared with acute phase, p < 0.05) at the convalescent phase in the GMPP group. These suggest that serum levels of osteocalcin and MMP-2 are impaired during MPP.

### 3.4 Cytokines that correlates with disease severity of SMPP

**Figure 8:**
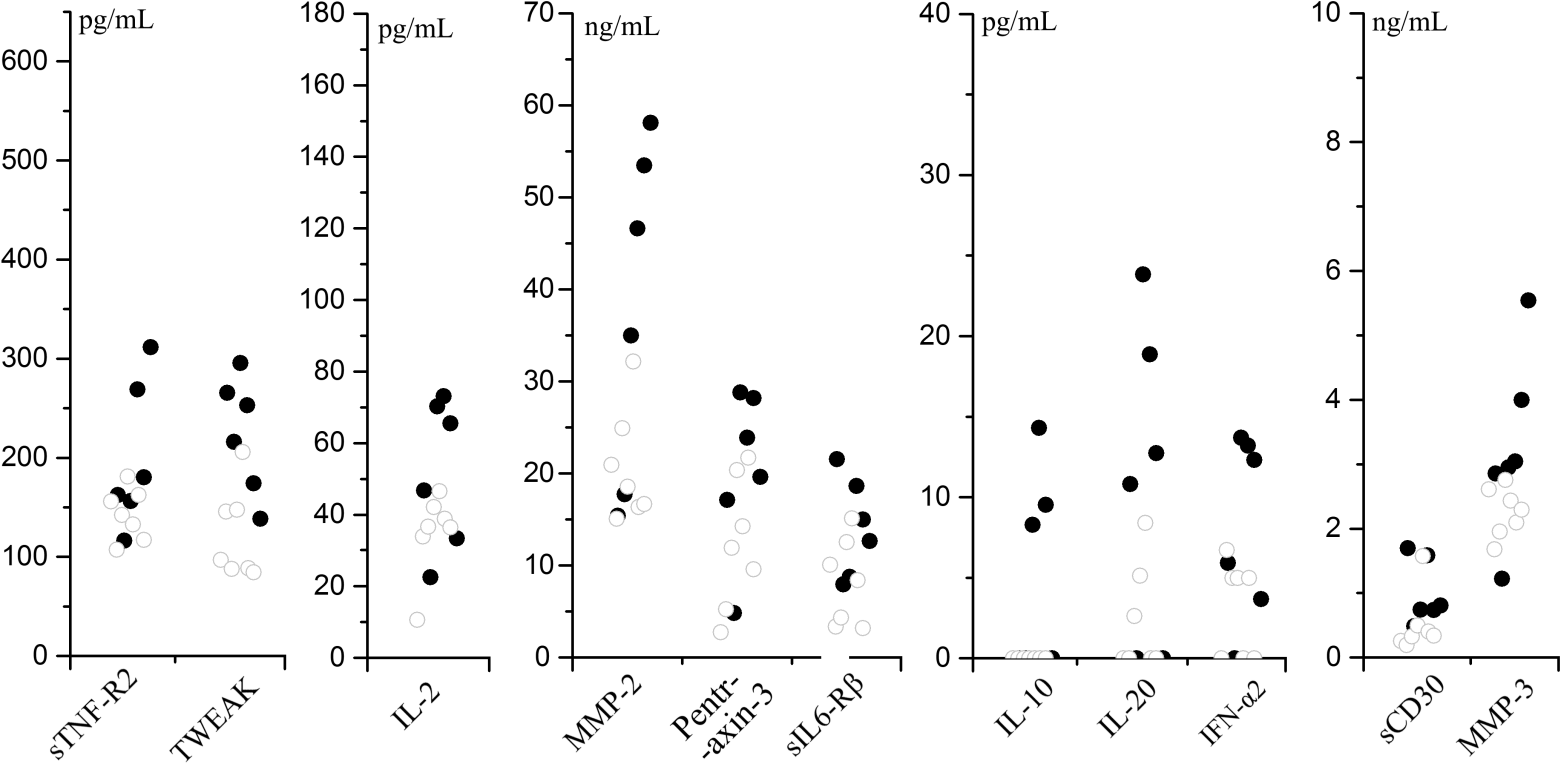
Cytokines that negative correlate to the application of bronchial lavage (p < 0.1) in SMPP group.

To analyze the correlation between cytokines and disease severity of SMPP, the application of bronchial lavatory was used to represent disease severity in the lung. As indicated, 11 cytokines (IL-2, IL-10, IL-20, IFN-α, sIL6-Rβ, Pentraxin-3, TWEAK, sTNF-R2, sCD30, MMP-2, MMP-3) in the SMPP group showed negative correlations to the application of bronchial lavage (p < 0.1) (**Figure 8**; **Table S3**). The levels of these cytokines may serve as biomarkers for the disease severity of SMPP.

## 4 Discussion

In the present study, our results suggest a stronger and better controlled immune response in GMPP than in SMPP. This is consistent with the lower proportion of lymphocytes and deduced immune disorder in the lung during SMPP (5, 25). Quick proliferation of MP would be an important reason for the development of SMPP, just as detected in BALF (15). The MP proliferation rate in the lung may relate to the site of infection; bronchial infections with MP easily cause local injury and exposure to blood, which in turn promote the proliferation of MP (29). Furthermore, a higher load of MP in the lung may lead to an increase in pro-inflammatory cytokines because the induction of IL8 has been demonstrated as dose-dependent *in vitro* (8). Elevated IL8 can enhance neutrophil infiltration. Higher proportions of neutrophils were detected in the BALF of SMPP patients (14). In mice, neutrophils have been proved to mediate lung injury during the infection of MP (30). In a previous study, we found that both cell damage and neutrophils contribute to MP infection and cytokine expression (31). Thus, a quick proliferation rate of MP may induce strong innate inflammatory responses before sufficient activation of adaptive immune responses, just as a higher rate of neutrophils and a lower rate of lymphocytes were detected in SMPP than in GMPP (**Table 1**). Clinically, antibiotics and herbal medicines have been shown to be effective in the treatment of MPP, which may relate to their inhibitory effects on MP proliferation and the induction of pro-inflammatory cytokines, respectively (32).

In addition, different immune responses during SMPP may be related to the basic immune status of the host. Repeated infection or avirulent vaccination can both induce exacerbated disease in mouse models (33, 34). The correlation between MP infection and asthma in children was also reported (35). Different pro-inflammatory cytokines in the lung may be induced under a hyperimmune state, for MP has been reported with strong IL-6 and IL-4 induction ability (36, 37), which may promote the differentiation of Th2 cells (10, 38). In the present study, the elevation of OPN, IL-26 and LIGHT may relate to T cell subset regulations during SMPP. OPN can enhance the expression of IL-12 and IFN-γ and reduce the expression of IL-10, thus promoting type I immunity (39). The level of OPN is associated with pulmonary inflammatory responses, and the expression of OPN increased in allergy, asthma, acute respiratory distress syndrome, fibrosis, parasitic infections, and *Klebsiella pneumoniae* pneumonia (40, 41). No associated study of OPN during MPP has been reported; our results suggest a positive role of OPN during SMPP for its negative correlation with lavatory treatment (p = 0.108). IL-26 is associated with the activation of Th17 and CD4^+^ IL-22^+^ T-cells (42, 43); it also plays a role in inflammatory bowel disease, which may associate with the extra-pulmonary gastroenteritis manifestation during SMPP (44). In addition, LIGHT, a tumor necrosis factor (TNF) superfamily ligand, can also promote T cell proliferation and activate the function of cytotoxic T-cells (45). In influenza virus (IAV)-induced pneumonia, a significant reduction in morbidity and mortality was observed in LIGHT knockout mice, suggesting that the increase of LIGHT may be associated with lung injury during SMPP (20, 46). LIGHT can also regulate TSLP to promote the development of pulmonary fibrosis, which is a common complication of SMPP (47). The correlations of OPN, IL-26, and LIGHT to T cell subset regulation during MPP have not been described, which needs further investigation.

Our results indicated potential immunosuppressive mechanisms during the infection of MP and the decrease of sIL-6Rβ and MMP-2. IL-6Rβ is known as GP130; it is the common receptor IL-11, leukemia inhibitory factor, oncostatin M, ciliary neurotrophic factor, and cardiotrophin-1. The absence of IL-6Rβ can lead to immune deficiency (48). In pneumonia caused by *Staphylococcus aureus*, the block of downstream pathways of IL-6Rβ reduces the clearance of *Staphylococcus aureus* (49). sIL-6Rβ in serum can antagonize the inflammatory response induced by IL-6 (50). A decrease of sIL-6Rβ may lead to the dysfunction of IL-6 with a potential contribution to the development of pneumonia (9). The level of sIL-6Rβ was reported decreased in patients with respiratory distress due to COVID-19 infection, exhibiting a lesser degree of reduction as displayed in this study (51). The decrease of IL-6Rβ may also influence the function of other cytokines like IL-11, reducing the activation macrophages and B cells as displayed in the present study (52). Transcriptome analysis of BALF in children with SMPP suggested the immunodeficiency of B cells (53), which may influence the pulmonary clearance of MP (54). In addition, IL-6Rβ functions in osteoblast maintenance, and decreases in IL-6Rβ may affect osteoblast activity, leading to decrease in osteocalcin (55). On the other hand, MMP-2 was also supposed to initiate primary innate immune responses by activating NF-κB, NFAT, and IRF (56). MMP-2 in blood has also been reported to be significantly reduced during MP infection in adults (57), but not in other CAP infections, suggesting a specific response to MP infections. The reduction of sIL-6Rβ and MMP-2 may be mediated by MP infection and influences the immune responses of the host, which needs further investigation.

The 16 cytokines with significant differences between SMPP and GMPP at the acute phase may serve as indicators of SMPP, and the combination of cytokines associated with macrophages (CHI3L1), B cells (BAFF), and T cells (IL-12, OPN, and IL-26) may serve as immune response pattern indicators for SMPP and GMPP. In addition, our data suggest the skin inflammation related TEWAK as a potential indicator of SMPP (58). The ratios of IL-2/TEWAK and IL-12/TEWAK are significantly different between GMPP and SMPP groups (**Table S3**). Up to now, several cytokines have been reported to correlate with SMPP, including low transcription level of IL-17A (59); high level of IL-4 and IL-6 in the lung; and high level of IL-5, IL-10, and IFN-γ in serum (60). The regulations of these cytokines do not agree with the results in this study; this may relate to the time point of sample collection. As displayed in the present study, cytokines in patients with GMPP mostly reach high levels at acute phase and decrease at convalescent phase, whereas lower elevation of multiple cytokines are detected at the acute phase and several of which elevated at the convalescent phase during SMPP, thus differential results may be obtained at different sample time points.

In conclusion, serum cytokine profiling reveals that the immune response patterns of GMPP and SMPP are different at acute phase. The degree of immune responses and activation patterns of T-cells, B-cells, and macrophages may associate with the development of MPP and serve as an indicator of GMPP and SMPP. This study gives a general view of the immune response patterns of GMPP and SMPP, which is helpful in understanding the pathogenesis of MPP.

## Data availability statement

The raw data supporting the conclusions of this article will be made available by the authors, without undue reservation.

## Ethics statement

The studies involving human participants were reviewed and approved by Bioethics Committee of Beijing Friendship Hospital Affiliated to Capital Medical University. Written informed consent to participate in this study was provided by the participants’ legal guardian/next of kin.

## Author contributions

ZZ, HD, DX and BX involved in conception, design, data analysis, drafting and reviewing the manuscript; PT and DS involved in data acquisition; RaW and RuW involved in sample preservation and clinical information collection; all other authors involved in sample collection and conception. All authors contributed to the article and approved the submitted version.
