# Supplementary material for: Serum cytokine profiling reveals different immune response patterns during general and severe Mycoplasma pneumoniae pneumonia
Source: Front Immunol. 2022 Dec 22;13:1088725. doi: 10.3389/fimmu.2022.1088725 (PMC9813340; doi:10.3389/fimmu.2022.1088725)
Supplement: Supplementary file 1 [file Table_1.docx]

**Table S1:** The level (mean±STD, pg/mL) of 37 kinds of cytokine in health group (Control), general MPP (GMPP) and severe MPP (SMPP) group at acute phase (AP) and recovery phase (RP).

|  | **Control** | **GMPP-AP** | **SMPP-AP** | **GMPP-RP** | **GMPP-RP** |
| --- | --- | --- | --- | --- | --- |
| IL-27 | 28.15±22.62 | 106.83±28.92**^C^** | 86.01±42.72**^C^** | 46.04±36.08**^A^** | 66.05±36.85**^C^** |
| IL-29(IFN-1) | 9.88±15.52 | 80.45±22.47**^C^** | 65.10±37.07**^C^** | 25.46±16.96**^A^** | 45.19±30.42**^C^** |
| sIL-6Rβ  (gp130) | 18496.68±4447.78 | 12829.73±5001.68**^C^** | 10878.26±5729.55**^C^** | 13656.34±5193.23**^C^** | 9803.44±5119.37**^C^** |
| APRIL  /TNFSF13 | 10241.81±6359.77 | 21296.84±3287.72**^C^** | 21772.04±6984.65**^C^** | 16126.37±5195.53**^C^** | 20411.72±7549.74**^C^** |
| Osteocalcin | 3838.64±794.33 | 1642.48±801.28**^C^** | 1714.32±1035.06**^C^** | 1915.88±784.67**^C^** | 1032.84±523.95**^C/G^**^/^**^A^** |
| Pentraxin-3 | 6243.74±2494.99 | 18455.18±8493.34**^C^** | 16012.43±8744.01**^C^** | 7830.40±3722.44**^A^** | 9579.25±7469.07**^A^** |
| MMP-2 | 134827.75±44548.87 | 44573.49±44400.53**^C^** | 28523.48±15294.96**^C^** | 50543.23±38415.15**^C^** | 27052.11±11671.94**^C^** |
| MMP-3 | 1751.14±623.90 | 3031.79±571.05**^C^** | 2724.67±1092.80**^C^** | 2167.49±802.40**^A^** | 2951.59±1428.26 |
| IL-2 | 13.18±8.58 | 58.31±11.11**^C^** | 42.80±18.09**^C/G^** | 27.06±7.71**^C/A^** | 30.59±9.51**^C/A^** |
| IL-10 | 0.00±0.00 | 6.69±5.83**^C^** | 2.47±4.87**^C/G^** | 0.00±0.00**^A^** | 0.26±0.94 |
| IL-11 | 3.75±1.44 | 11.21±2.95**^C^** | 7.49±2.87**^C/G^** | 5.25±1.79**^A^** | 5.42±2.32**^A^** |
| IL-12(p40) | 65.57±19.65 | 161.26±41.43**^C^** | 109.30±36.81**^C/G^** | 79.76±26.21**^A^** | 87.47±29.88**^C^** |
| IL-20 | 0.00±0.00 | 15.57±6.99**^C^** | 6.34±8.08**^C^** | 1.09±1.77**^A^** | 1.99±4.19 |
| IL-28A | 30.56±23.79 | 108.69±27.14**^C^** | 76.05±39.11**^C/G^** | 50.85±25.58**^A^** | 51.14±27.96**^A^** |
| IL-32 | 38.57±17.98 | 85.68±21.72**^C^** | 65.58±29.27**^C/G^** | 46.41±14.10**^A^** | 49.73±17.11 |
| IL-35 | 184.92±84.88 | 471.74±111.91**^C^** | 316.89±167.71**^C/G^** | 218.00±79.99**^A^** | 211.54±113.81**^A^** |
| TSLP | 6.52±2.33 | 14.47±3.85**^C^** | 11.48±3.71**^C/G^** | 7.72±2.15**^A^** | 8.17±3.42**^A^** |
| IFN-α2 | 0.41±1.43 | 11.57±3.73**^C^** | 5.42±4.99**^C/G^** | 1.16±2.85**^A^** | 1.94±3.20**^A^** |
| IFN-β | 7.75±5.99 | 22.82±3.81**^C^** | 16.43±8.01**^C/G^** | 13.09±3.75**^C/A^** | 12.95±5.78**^C^** |
| IFN-γ | 63.16±19.83 | 120.97±20.95**^C^** | 99.31±24.88**^C/G^** | 80.80±12.90**^C/A^** | 81.63±26.95**^C/A^** |
| IL-8 | 13.28±12.96 | 25.56±10.37**^C^** | 18.41±15.11 | 21.96±16.90 | 15.37±12.72 |
| BAFF | 3259.06±756.40 | 6344.45±2715.43**^C^** | 4188.99±1901.83^G^ | 3656.34±1356.44**^A^** | 2365.27±1311.85**^A^** |
| CHI3L1 | 2979.40±1298.06 | 1825.75±721.44**^C^** | 2917.55±1456.46^G^ | 2416.58±1246.71 | 2510.80±1270.39 |
| TWEAK | 179.76±46.03 | 122.59±50.93 | 169.14±72.60**^C^** | 169.52±71.44 | 197.44±86.41 |
| IL-26 | 460.52±247.89 | 650.45±204.53 | 751.34±354.21**^C^** | 381.27±222.74**^A^** | 767.75±367.45**^C^** |
| Osteopontin  (OPN) | 7833.72±3392.58 | 6693.95±3006.67 | 12994.70±9883.42**^C/G^** | 5185.93±2254.91 | 11636.04±8417.58 |
| LIGHT | 0.00±0.00 | 2.34±4.25 | 8.15±9.62**^C/G^** | 4.70±9.78 | 2.49±7.90**^A^** |
| sIL-6Rα | 886.06±385.63 | 1872.76±2015.94 | 2710.86±2689.16 | 1610.98±1773.88 | 1385.67±1185.16 |
| sCD163 | 39373.76±18675.64 | 35662.63±15895.35 | 38222.47±18169.87 | 40995.00±16584.03 | 35986.16±17121.19 |
| sCD30 | 366.56±253.15 | 596.44±262.80 | 741.58±535.03 | 676.22±648.09 | 454.63±248.11 |
| sTNF-R1 | 336.96±105.15 | 380.13±65.51 | 352.67±193.06 | 350.39±100.25 | 278.07±214.54 |
| sTNT-R2 | 147.67±32.93 | 165.50±25.13 | 168.71±59.34 | 149.32±51.01 | 135.14±51.64 |
| MMP-1 | 651.35±473.86 | 884.93±187.63 | 924.91±362.72 | 983.51±442.59 | 801.97±674.06 |
| IL-12(p70) | 0.00±0.00 | 0.00±0.00 | 0.00±0.00 | 0.00±0.00 | 0.00±0.00 |
| IL-22 | 0.00±0.00 | 0.00±0.00 | 2.62±9.07 | 3.62±11.45 | 0.00±0.00 |
| IL-34 | 93.80±136.54 | 58.02±94.89 | 124.03±175.05 | 94.34±210.19 | 67.55±179.04 |
| IL-19 | 4.99±7.10 | 9.47±15.50 | 11.33±13.43 | 8.19±11.71 | 7.73±12.43 |

“**^C^**” indicate significant difference to Control group; “**^G^**” indicate significant difference to to GMPP group at acute phase; “**^G^**” indicate significant difference to GMPP group at recovery phase; “**^A^**” indicate significant difference to acute phase within groups.

Table S2: Information of the participants in general MPP (GMPP) and severe MPP (SMPP) group.

| GMPP | | | | SMPP | | | |
| --- | --- | --- | --- | --- | --- | --- | --- |
| ID | Gender | Age | BAL* | ID | Gender | Age | BAL* |
| BT27/28 | male | 3.11 | No | BT31/32 | male | 3.8 | No |
| BT11/12 | male | 5 | No | ETBX380/381 | male | 4.6 | No |
| BT23/24 | male | 5.2 | No | ETBX404/405 | male | 5.5 | Yes |
| BT33/34 | male | 6 | No | ETBX382/383 | male | 6 | No |
| BT5/6 | male | 7 | No | ETBX391/392 | male | 6.5 | No |
| BT25/26 | male | 8 | No | BT35/36 | male | 7 | Yes |
| BT41/42 | male | 8 | No | ETBX397/398 | male | 7.1 | No |
| BT13/14 | female | 5 | No | ETBX360/362 | male | 8 | Yes |
| BT43/44 | female | 7 | No | ETBX374/376 | female | 7 | Yes |
| BT39/40 | female | 8 | No | ETBX384/385 | female | 9 | No |
| BT3/4 | female | 9 | No | BT37/38 | female | 9 | Yes |
|  |  |  |  | ETBX393/395 | female | 9.1 | Yes |
|  |  |  |  | ETBX386/388 | female | 10 | Yes |

* “BAL” indicated application of bronchoalveolar lavage

Table S3: The ratios of cytokines that potentially applied for distinguish of general MPP (GMPP) and severe MPP (SMPP) group.

|  | Group | average±STD | Range | P value* |
| --- | --- | --- | --- | --- |
| IL2/TWEAK | Control | 0.0713±0.0366 | 0-0.1275 | C/G=0.001 |
|  | GMPP | 0.5747±0.283 | 0.1844-1.2899 | G/S=0.014 |
|  | SMPP | 0.2742±0.1093 | 0.1041-0.4391 | S/C=0.001 |
| IL12/TWEAK | Control | 0.3794±0.1268 | 0.2085-0.6266 | C/G=0.004 |
|  | GMPP | 1.6367±0.9391 | 0.2764-4.0759 | G/S=0.021 |
|  | SMPP | 0.7322±0.3334 | 0.2806-1.3687 | S/C=0.011 |

* “C” short for Control; “G” short for GMPP; “S” short for SMPP
